# Risk stratification of postoperative cardiopulmonary toxicity after trimodality therapy for esophageal cancer

**DOI:** 10.3389/fonc.2023.1081024

**Published:** 2023-02-09

**Authors:** Roman O. Kowalchuk, Grant M. Spears, Lindsay K. Morris, Dawn Owen, Harry H. Yoon, Krishan Jethwa, Michael D. Chuong, Matthew J. Ferris, Michael G. Haddock, Christopher L. Hallemeier, Dennis Wigle, Steven H. Lin, Kenneth W. Merrell

**Affiliations:** ^1^ Department of Radiation Oncology, Mayo Clinic, Rochester, MN, United States; ^2^ Department of Statistics, Mayo Clinic, Rochester, MN, United States; ^3^ Department of Medical Oncology, Mayo Clinic, Rochester, MN, United States; ^4^ Department of Radiation Oncology, Miami Cancer Institute, Miami, FL, United States; ^5^ Department of Radiation Oncology, University of Maryland Medical System, Baltimore, MD, United States; ^6^ Department of Thoracic Surgery, Mayo Clinic, Rochester, MN, United States; ^7^ Department of Radiation Oncology, MD Anderson Cancer Center, Houston, TX, United States

**Keywords:** trimodality therapy, cardiac toxicity, radiotherapy toxicity, modeling, outcomes, esophagus

## Abstract

**Purpose/objective:**

Postoperative toxicity for esophageal cancer impacts patient quality of life and potentially overall survival (OS). We studied whether patient and toxicity parameters post-chemoradiation therapy predict for post-surgical cardiopulmonary total toxicity burden (CPTTB) and whether CPTTB was associated with short and long-term outcomes.

**Materials/methods:**

Patients had biopsy-proven esophageal cancer treated with neoadjuvant chemoradiation and esophagectomy. CPTTB was derived from total perioperative toxicity burden (Lin et al. JCO 2020). To develop a CPTTB risk score predictive for major CPTTB, recursive partitioning analysis was used.

**Results:**

From 3 institutions, 571 patients were included. Patients were treated with 3D (37%), IMRT (44%), and proton therapy (19%). 61 patients had major CPTTB (score ≥ 70). Increasing CPTTB was predictive of decreased OS (p<0.001), lengthier post-esophagectomy length of stay (LOS, p<0.001), and death or readmission within 60 days of surgery (DR60, p<0.001). Major CPTTB was also predictive of decreased OS (hazard ratio = 1.70, 95% confidence interval: 1.17-2.47, p=0.005). The RPA-based risk score included: age ≥ 65, grade ≥ 2 nausea or esophagitis attributed to chemoradiation, and grade ≥ 3 hematologic toxicity attributed to chemoradiation. Patients treated with 3D radiotherapy had inferior OS (p=0.010) and increased major CPTTB (18.5% vs. 6.1%, p<0.001).

**Conclusion:**

CPTTB predicts for OS, LOS, and DR60. Patients with 3D radiotherapy or age ≥ 65 years and chemoradiation toxicity are at highest risk for major CPTTB, predicting for higher short and long-term morbidity and mortality. Strategies to optimize medical management and reduce toxicity from chemoradiation should be strongly considered.

## Introduction

Trimodality therapy for esophageal cancer consists of concurrent chemoradiotherapy followed by esophagectomy and is the standard of care for patients with resectable, non-metastatic stage 2 or greater disease (1–3). Side effects associated with trimodality therapy, however, can substantially impact patient quality of life. Though the rates of grade ≥ 3 (G3+) toxicity post-chemoradiotherapy were low in the CROSS trial (including 5% anorexia and 6% leukopenia), at least 30% of patients experienced any grade anorexia, fatigue, nausea, leukopenia, and thrombocytopenia. Further, 46% of patients experienced post-operative pulmonary complications in the neoadjuvant chemoradiotherapy arm, and 21% of patients suffered post-operative cardiac complications, with postoperative morality of 4% (4). The reported lengths of hospital stay post-esophagectomy (LOS) vary, but post-operative complications are also key drivers of post-operative readmission rates within 30-90 days after esophagectomy (5–7). Some studies also report potential correlations between increased LOS and reduced OS (8, 9).

Efforts to reduce the burden of toxicity have primarily consisted of optimizing radiotherapy dose delivery, supportive care, and selective esophagectomy (10–12). The underlying hypothesis considers the possibility that neoadjuvant chemoradiotherapy influences post-operative toxicity. For instance, reduction of heart and lung irradiation with advanced radiotherapy techniques may be associated with a lower risk of cardiac toxicity (13). A recent randomized clinical trial showed that a reduction in irradiation of organs at risk resulted in less severe adverse events with use of proton beam radiotherapy relative to intensity modulated radiotherapy, which currently is the topic of the NRG GI006 clinical trial (12). Proton beam radiotherapy can also reduce the risk of hematologic toxicity, such as severe lymphopenia (14). Though additional studies are undoubtedly required, selective esophagectomy is another strategy that has demonstrated encouraging OS in carefully selected patients and may provide the opportunity to reduce treatment-related morbidity (15, 16). Other investigators have explored prehabilitation to optimize a patient’s status prior to surgery, in the hopes of reducing post-operative complications (17–19).

In this study, we aimed to evaluate a new composite variable, post-operative cardiopulmonary total toxicity burden (CPTTB), for its predictive power for OS, LOS, and death or readmission within 60 days of surgery (DR60) (12). We also sought to investigate whether toxicity post-chemoradiotherapy is predictive of post-operative complications to determine which patients might benefit from additional pre-operative management or even omission of esophagectomy.

## Methods

### Data collection

Further details and inclusion and exclusion criteria concerning this data have been previously published (20). Briefly, 571 patients treated with trimodality therapy for biopsy-proven, American Joint Committee on Cancer 7^th^ edition staging system cT1N1-3M0 or cT2-4aN0-3M0 esophageal cancer were included from 3 academic institutions (21). Consecutive patients treated at these 3 institutions between 2007 through 2013 were included. Trimodality therapy consisted of neoadjuvant concurrent chemoradiotherapy followed by esophagectomy. Induction chemotherapy was allowed but not required, but all patients in this dataset were required to have proceeded to esophagectomy. Multidisciplinary evaluation of all patients was required. Follow-up was conducted per institutional standards; however, at least 2 years of follow-up was required for survivors after surgery for inclusion in any survival analyses to minimize mortality bias. Most commonly, patients had scheduled follow-up every 3 months for the first year, every 6 months for the next 2 years, and then subsequent annual follow-ups. Physical examination and CT or PET/CT were standard at all follow-up visits, but utilization of surveillance esophagogastroduodenoscopy was variable. Toxicities were distinguished between toxicity during or post-chemoradiotherapy (but prior to esophagectomy) and post-operative side effects. Adverse effects were graded using the Common Terminology Criteria for Adverse Events version 4.0. Institutional review board approval was obtained at all participating institutions.

### Treatment details

Induction chemotherapy was allowed and generally consisted of 4-8 weeks of therapy with a fluoropyrimidine, a platinum agent, and/or a taxane when utilized (22). Concurrent chemotherapy agents were variable among the participating institutions (**Table 1**). The median radiotherapy dose was 50.4 Gy in 28 fractions delivered 5 days weekly using 3D conformal radiotherapy, intensity modulated radiation therapy (IMRT), or proton beam therapy. Esophagectomy consisted of Ivor-Lewis, other transthoracic, transhiatal, or other surgical approaches. Surgical technique was selected by the operating team. All patients underwent preoperative staging approximately 4-6 weeks after completing neoadjuvant therapy.

**Table 1:** Patient characteristics are demonstrated.

Patient characteristic	Number (%)*
Age at diagnosis (years, IQR)	60 (IQR: 53-67)
Gender	
Male	486 (85%)
Female	85 (15%)
Underlying comorbidities	
History of heavy (≥ 6 drinks/day) alcohol use	86 (15%)
Coronary artery disease	75 (13%)
Diabetes	92 (16%)
COPD	41 (7%)
Tumor characteristics	
Adenocarcinoma	530 (93%)
Squamous cell carcinoma	40 (7%)
Poor tumor differentiation**	362 (65%)
Moderate tumor differentiation	192 (34%)
Well-differentiated tumor	6 (1%)
Median tumor length (cm, IQR)	5 (IQR: 3-7)
Extension into stomach	228/538 (42%)
Clinical staging	
I	4 (1%)
IIa	160 (28%)
IIb	41 (7%)
III	343 (61%)
IVa	15 (3%)
IVb	3 (1%)
N/A	5 (1%)
Treatments	
Induction chemotherapy	139 (24%)
3D conformal radiotherapy	211 (37%)
IMRT	251 (44%)
Proton beam radiotherapy	109 (19%)
Median dose (Gy, IQR)	50.4 (IQR: 50.4-50.4)
Median fractions (IQR)	28 (IQR: 28-28)
Concurrent chemotherapy	
Cisplatin/5-FU	138 (24%)
Carboplatin/paclitaxel	87 (15%)
Oxaliplatin/5-FU	148 (26%)
5-FU/docetaxel	142 (25%)
DFOX	21 (4%)
Other	35 (6%)
Surgical information	
Days to surgery (from completion of chemoRT, IQR)	49 (IQR: 42-63)
Ivor Lewis	479 (84%)
Transthoracic	19 (3%)
Other procedures	73 (13%)
Median lymph nodes removed (IQR)	20 (IQR: 15-27)
Median lymph node positive (IQR)	0 (IQR: 0-1)
Pathologic complete response	179 (31%)

IQR, interquartile range; COPD, chronic obstructive pulmonary disease; IMRT, intensity modulated radiation therapy; 5-FU, fluorouracil; DFOX, docetaxel, folinic acid, fluorouracil, and oxaliplatin; chemoRT, chemoradiotherapy.

*Numbers in parentheses indicate the percentage incidence, unless otherwise stated.

**Only 560 patients had tumor differentiation available for assessment.

### Endpoints and statistical analysis

The principal endpoints of our analysis were CPTTB and OS. OS was defined as the time from surgery to death or loss to follow-up. CPTTB is a new metric derived from the work of Lin et al. (12). The 5 most frequent post-operative cardiopulmonary toxicities (myocardial infarction, non-ischemic cardiac, pleural effusion, pneumonia, ARDS) were used to create an aggregate CPTTB score. Weights were determined as “best opinion” per the previous work of Lin et al., which more heavily weighted more severe toxicity (e.g. myocardial infarction) relative to generally less severe side effects (e.g. pleural effusion) (**Table 2**) (12). The CPTTB score is derived as the sum of the weights of each individual cardiopulmonary toxicity. All components available from the initial assessment by Lin et al. were included within our analysis.

**Table 2:** The components of cardiopulmonary total toxicity burden (CPTTB) are tabulated, along with their corresponding weights.

CPTTB component	Weight
Myocardial infarction	70
Non-ischemic cardiac toxicity	30
Pleural effusion	30
Pneumonia	40
Acute respiratory distress syndrome	90

CPTTB, cardiopulmonary total toxicity burden.

A CPTTB risk score was developed using RPA to predict for major CPTTB. Major CPTTB was defined as CPTTB score greater than or equal to 70 a-priori, corresponding to a major post-operative toxicity (myocardial infarction or ARDS) or multiple intermediate post-operative toxicities. We aimed to first assess the predictive and prognostic power of CPTTB; then we sought to generate a CPTTB risk score predictive of major CPTTB. Associations between CPTTB, as both a continuous and categorical variable, and OS were assessed using Cox proportional hazards analysis. The CPTTB risk score was generated and used to predict CPTTB as both a continuous and discretized outcome using RPA. Secondary endpoints included LOS and DR60. Disease-free survival considered the time to recurrence or death (from any cause) while disease-specific survival only included death if it was secondary to disease (as opposed to another cause). DR60 was analyzed as a binary event using logistic regression, and LOS as a continuous outcome using linear regression. Statistical significance was defined in all instances as p < 0.05, and 95% confidence intervals (CI) were recorded throughout the analysis. Statistical analyses were performed using SAS, version 9.4, and R, version 3.6.2.

### Recursive partitioning analysis

RPA was utilized in an effort to develop a CPTTB risk score predictive of major CPTTB. This approach incorporated decision-tree analysis, and this methodology has been previously utilized and published (23–25). RPA was conducted in Python (v3.8.0) using open-source packages (26–28). The binary endpoint of major CPTTB was used as the endpoint of RPA. Node-splitting required a minimum of 15 patients in each group, but a range of 15-20 was explored. RPA trees were only allowed to consist of two levels, such that there were a maximum of 4 groups. Prior to model generation, a correlation heatmap was generated to assess for correlation between candidate variables, and feature importance testing was conducted to minimize overfitting. All candidate RPA models were developed using the training set (60%) and refined with an internal validation set (20%), with accuracy subsequently assessed through an independent test set (20%). This approach allowed for consideration of potential correlations between candidate variables, as well as internal validation. Variables used in the highest-fidelity models were then considered for incorporation in the resulting risk score.

## Results

### Dataset

The final dataset consisted of 571 patients from 3 institutions with demographic and tumor features shown in **Table 1**. The median age at diagnosis was 60 years (interquartile range [IQR]: 53-67), with a predominance of male patients (85%). Most tumors were adenocarcinoma (93%), with 65% poorly differentiated. Almost all cases involved distal esophageal tumors (93%) and most were staged as IIa (28%) or III (60%). Median radiation dose was 50.4 Gy (IQR: 50.4-50.4), and median fractionation number was 28 (IQR: 28-28), with 89% of patients treated with 28 fractions delivering 50.4 Gy. Radiotherapy treatment modalities include 3D conformal (37%), IMRT (44%), and proton therapy (19%).

### Overall toxicity

Treatment-related adverse events were common both post-chemoradiotherapy and post-operatively (**Table 3**). A total of 14% of patients required a feeding tube, and G2+ dysphagia, esophagitis, nausea, and fatigue each occurred in at least 30% of patients during or immediately after chemoradiotherapy. The most common post-operative toxicities were non-ischemic cardiac toxicity (16%), pleural effusion (13%), and pneumonia (13%). Non-ischemic cardiac toxicity most often involved atrial fibrillation and acute exacerbation of congestive heart failure. The median length of hospital stay post-operatively was 9 days (IQR: 7-13) and 19% of patients had DR60: death or readmission to the hospital within 60 days of esophagectomy.

**Table 3:** Treatment related adverse events.

Toxicity	Number (%)*
Chemoradiotherapy AE	
Median weight loss (% body weight, IQR)	4 (IQR: 1-8)
Feeding tube placed	80 (14%)
G2+ dysphagia	172 (30%)
G2+ esophagitis	258 (45%)
G2+ nausea	266 (47%)
G2+ fatigue	184 (32%)
G2+ anorexia	88 (15%)
G2+ hematologic	116 (20%)
G3+ hematologic	51 (9%)
Post-operative AE	
Myocardial infarction	6 (1%)
Non-ischemic cardiac toxicity	93 (16%)
Pleural effusion	74 (13%)
Pneumonia	75 (13%)
ARDS	28 (5%)
Toxicity-related outcomes	
DR60	111 (19%)
Median LOS (days, IQR)	9 (IQR: 7-13)

AE, adverse event; IQR, interquartile range; G2+, ≥ grade 2; G3+, ≥ grade 3; ARDS, acute respiratory distress syndrome; DR60, death or readmission within 60 days after esophagectomy; LOS, length of hospital stay after esophagectomy.

*Numbers in parentheses indicate the percentage incidence, unless otherwise stated.

### Cardiopulmonary total toxicity burden

**Figure 1:**
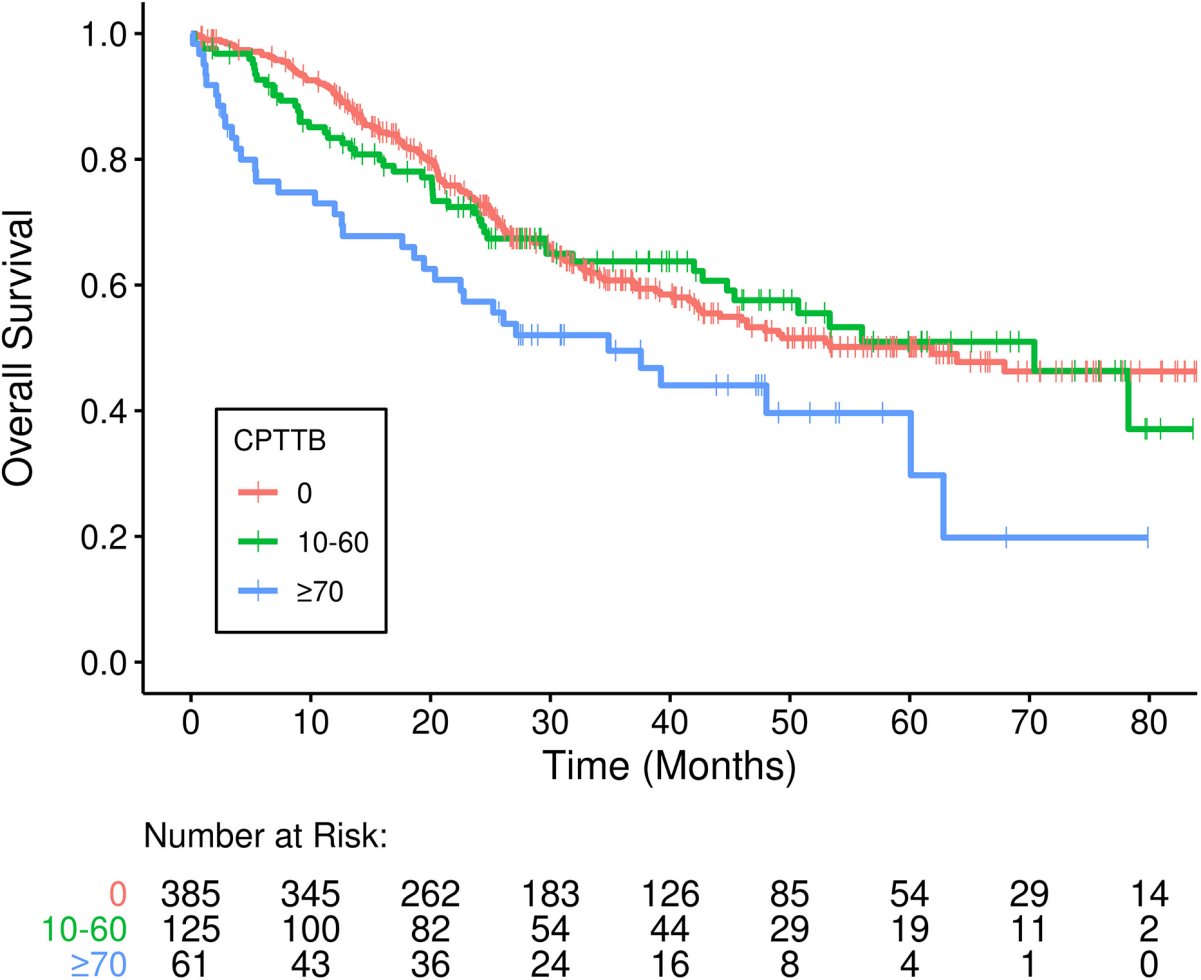
Inferior overall survival (OS) is demonstrated in patients who developed major cardiopulmonary total toxicity burden (CPTTB) (≥ 70) (p=0.005).

As a continuous variable, CPTTB was predictive of decreased OS (hazard ratio [HR] = 1.07 per 10-point increase in CPTTB, 95% confidence interval [CI]: 1.03-1.10, p<0.001). CPTTB was discretized into major CPTTB (CPTTB score ≥ 70), minor CPTTB (0 < CPTTB ≤ 60), and no CPTTB. Though only 11% of patients had major CPTTB, a statistically significant association of major CPTTB with decreased OS (HR = 1.70, 95% CI: 1.17-2.47, p=0.005) was demonstrated (**Figure 1**).

**Figure 2:**
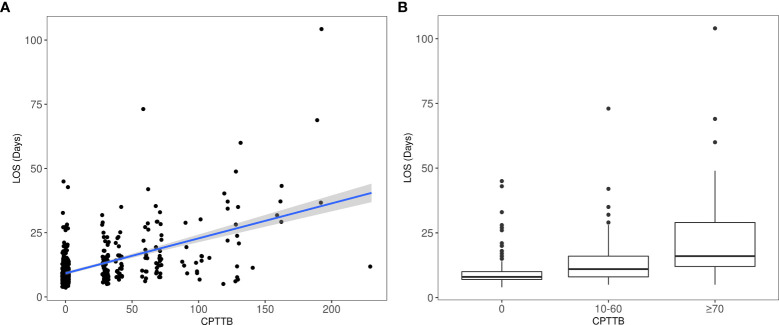
Increased length of hospital stay after esophagectomy (LOS) is also demonstrated with major CPTTB (p<0.001), both as a continuous variable **(A)** and as a discretized variable **(B)**.

Secondarily, CPTTB was shown to be predictive of increased DR60 and LOS. As a continuous variable, CPTTB was associated with increased risk of DR60 with an odds ratio (OR) of 1.25 (95% CI: 1.18-1.32, p<0.001) per 10-point increase in CPTTB. Major and minor CPTTB were associated with odds ratios of 10.3 (95% CI: 5.61-19.0, p<0.001) and 5.63 (95% CI: 3.40-9.31, p<0.001) for DR60. CPTTB was also associated with increased LOS as a continuous variable (p<0.001) (**Figure 2A**) and as a discretized variable (p<0.001) (**Figure 2B**).

### Predictors of CPTTB

Candidate pre-operative variables, including toxicity attributed to chemoradiotherapy, for prediction of major CPTTB were selected as those with p<0.10 *via* Wilcoxon p-value on univariate analysis (**Supplemental Table 1**). In total there were 61 patients with CPTTB ≥ 70, with a median CPTTB of 0 (range: 0-230). A correlation heatmap was then generated, revealing correlation between G2+ and G3+ hematologic toxicity, as well as the types of concurrent chemotherapy. 3D radiotherapy was also discarded secondary to significant correlation with post-chemoradiotherapy toxicity. Feature importance testing was then conducted, prior to model generation (**Supplemental Table 2**). RPA was performed with the remaining candidate variables. The highest-fidelity models demonstrated high degrees of accuracy in the validation (91%) and test (93%) sets, revealing that G2+ nausea, G2+ esophagitis, G3+ hematologic toxicity, and age ≥ 65 years were the most important variables in the highest-fidelity models. A resulting model was generated, with 1 point awarded for (a) age ≥ 65, (b) G2+ nausea and/or G2+ esophagitis attributed to chemoradiation, and (c) G3+ hematologic toxicity attributed to chemoradiation. Additional analyses of potential predictive factors for CPTTB are shown in **Supplemental Tables 3** and **4**. Of note, the delivery of induction chemotherapy did not result in increased CPTTB (**Supplemental Table 3**).

### Assessment of CPTTB risk score performance

A resulting model for the CPTTB risk score was generated. The CPTTB risk score effectively predicted the incidence of major CPTTB, as patients with a score of ≥ 2 had an 18% risk of major CPTTB, compared to 10% with a score of 1, and 2% with a score of 0 (**Table 4**). Scores of 2 and 3 were grouped together, as only 20 patients had a score of 3. The model demonstrated an AUC of 0.66, p<0.001. A score of 1 showed an OR of 5.15 (95% CI: 1.54-17.3, p=0.008), and a score of 2-3 showed OR of 9.78 (95% CI: 2.91-32.8, p<0.001) for major CPTTB. The CPTTB risk score was also predictive of continuous CPTTB (p<0.001). CPTTB risk scores of 1 and 2-3 were associated with an increase in CPTTB of 10.3 (95% CI: 3.1-17.6) and 22.1 (95% CI: 14.2-30.1) points, respectively, over a risk score of 0. The score directly predicted for DR60 (p=0.017) but not LOS (p=0.078). The risk score was not predictive of either disease-free survival (p=0.37) or disease-specific survival (p=0.73).

**Table 4:** The performance of the CPTTB risk score for predicting major CPTTB is tabulated below.

CPTTB risk score	Major CPTTB (%)	Odds ratio (95% CI)
Score: 0	3/136 (2%)	1.00 (reference)
Score: 1	28/269 (10%)	5.15 (95% CI: 1.54-17.3), **p<0.001**
Score: 2-3 Score: 2 Score: 3	30/166 (18%) 25/146 (17%) 5/20 (25%)	9.78 (95% CI: 2.91-32.8), **p<0.001**

CPTTB, cardiopulmonary total toxicity burden; 95% CI, 95% confidence interval.

Bold values indicate statistical significance.

### 3D radiotherapy

Though 3D radiotherapy was excluded as a candidate variable from model development due to significant correlation with post-chemoradiotherapy toxicity, it was assessed separately. Patients treated with 3D radiotherapy had worse OS (HR=1.40, 95% CI: 1.08-1.81, p=0.010) but not disease-free survival (HR=1.22, 95% CI: 0.96-1.54, p=0.098) when compared with patients treated with IMRT or proton beam radiotherapy (**Figure 2**). Of 211 patients treated with 3D radiotherapy, 39 (18.5%) experienced major CPTTB compared with only 6.1% of patients treated with IMRT or proton therapy (p<0.001). Patients treated with 3D radiotherapy also had increased LOS (mean 13.3 days vs. 10.9 days, p=0.002) and risk of DR60 (OR=1.67, 95% CI: 1.10-2.54, p=0.017).

The model demonstrated greater predictive power for major CPTTB (AUC=0.70) among patients treated with IMRT or proton therapy relative to those only treated with 3D radiotherapy (AUC=0.56). The model was also predictive of CPTTB as a continuous variable for all patients or only patients treated with IMRT or proton therapy (p<0.001 and p=0.001, respectively), but it was not predictive in patients treated with 3D radiotherapy (p=0.12). Finally, adherence to standard heart and lung dose constraints (both from Quantec and our clinical practice), as stratified by treatment modality, is shown in **Supplemental Table 5**.

## Discussion

In this analysis, we present a cohesive study of an surrogate variable for post-operative complications: CPTTB. We sequentially demonstrate that (1) CPTTB predicts for OS and multiple important secondary surgical endpoints, including LOS and DR60; (2) advanced age and chemoradiotherapy toxicity are key predictors for the development of major CPTTB; and (3) 3D radiotherapy portends reduced OS and increased CPTTB compared with more conformal techniques, such as IMRT and proton therapy. While the associated morbidity and impacts on patient quality of life secondary to post-operative toxicity have been previously reported, the association with OS is particularly striking and encourages additional study into optimizing pre-operative management, neoadjuvant chemoradiotherapy techniques, and patient selection for esophagectomy (5–7).

CPTTB represents a surrogate variable based upon the work of Lin et al. demonstrating the reduction of side effects with proton therapy compared to IMRT in a randomized phase IIB clinical trial (12). In the present analysis, we build upon this foundation by demonstrating a strong association between CPTTB and OS. For some patients, this reduction in OS could minimize or even eliminate the corresponding benefit derived from trimodality therapy (1, 2). Our analysis cannot definitively discern whether CPTTB predicts for patients who may have reduced OS secondary to post-operative toxicity or alternatively decreased OS due to other medical comorbidities; however, the fact that underling medical conditions (including coronary artery disease and diabetes mellitus) were generally not predictive of CPTTB suggests at least some element of a causative relationship. Notably, these results are best applied to distal esophageal adenocarcinomas.

Efforts to determine the impact of chemoradiotherapy toxicity on post-operative complications resulted in the development of the CPTTB risk score and revealed multiple factors predictive of major CPTTB, including advanced age, G3+ hematologic toxicity, and G2+ nausea and/or G2+ esophagitis. While our analysis cannot conclusively demonstrate the underlying reasons for this association, hematologic toxicity can increase the likelihood of infection, and pneumonia remains one of the principal post-operative complications after esophagectomy (29–31). Nausea and esophagitis could potentially impact post-operative caloric intake and malnutrition, thereby limiting post-operative recovery. The influence of age on post-operative complications remains controversial, as other reports have demonstrated minimal impact on outcomes (32, 33). It is possible that age serves as a surrogate for the presence of other medical comorbidities not directly assessed in our analysis. Even so, we present an RPA-based score predictive of post-esophagectomy cardiopulmonary toxicity, which could be used to further guide clinical decision-making by identifying patients who may benefit from further medical optimization prior to proceeding with surgical resection.

3D radiotherapy was an important predictor of both OS and CPTTB. Though patients treated with 3D radiotherapy had reduced OS, a statistically significant association with disease-free survival was not identified, suggesting that CPTTB likely has at least some direct impact on OS. An analysis by Ling et al. demonstrated the potential for a reduction in cardiopulmonary toxicity with proton beam radiotherapy in this setting, further supporting our conclusions (34). A systematic review and meta-analysis also derived a similar result: a reduction in dose delivered to the lungs and heart was identified with IMRT compared to 3D radiotherapy (35). Overall, our conclusions add to the growing body of literature that IMRT or proton beam radiotherapy should be preferred to 3D radiotherapy in the setting of trimodality treatment for esophageal cancer. Multiple studies have identified further benefits beyond only cardiopulmonary toxicity, including achieving lower vertebral bone marrow doses to reduce acute bone marrow toxicity (36, 37). In light of this recommendation, our predictive model for CPTTB was reassessed for only the subset of patients treated with IMRT or proton therapy, and higher accuracy was shown. This finding suggests that our model can continue to be utilized in the setting of IMRT and proton therapy.

Various approaches could be employed to minimize toxicity for patients at high risk for side effects. In the setting of esophagectomy, encouraging results using prehabilitation (optimization of a patient’s status prior to surgery) have been reported, prompting the development of prospective studies (17–19). A randomized trial of 68 patients with prehabilitation consisting of exercise and nutrition optimization demonstrated improved functional capacity before and after surgery compared to the control group (38). A recent network meta-analysis confirmed that, while neoadjuvant chemoradiotherapy improved OS compared to surgery or chemoradiotherapy alone, neoadjuvant chemoradiotherapy increased the risk of postoperative mortality (39). These results support the notion that trimodality therapy requires careful assessment of appropriate patient selection. Patients also require optimized medical management for symptoms, including pre-treatment antiemetics and consideration for proactive prescriptions of analgesics. Finally, in the recent EA2174 phase II/III study, elective nodal radiation was not recommended for patients with node-negative disease (40). Though omission of elective nodal volumes remains controversial in this setting, it may contribute to reduction of dose to organs at risk and thereby reduce the risk of side effects.

Though many institutions routinely prescribe 50-50.4 Gy in the setting of neoadjuvant chemoradiation, the dose could also be decreased to 41.4 Gy (per the CROSS trial) in an effort to reduce toxicity (1). In the CROSS trial, any grade esophagitis was only 19% (and only 1% grade 3 or higher esophagitis), substantially lower than in the current study (4). In the systematic review by Li et al., no detriment was identified with delivery of 41.4 Gy compared with higher doses, further supporting the potential use of dose deescalation in select patients (41). It is unknown whether the benefits of proton therapy and IMRT compared to 3D radiotherapy translate as strongly with the delivery of 41.4 Gy, as opposed to 50-50.4 Gy. A major benefit of prescription doses of 50-50.4 Gy is that if a patient does not proceed to surgical resection for any reason, then a definitive dose of chemoradiation was still delivered. For this reason, if omission of surgery is being considered for a given patient, prescription doses of 50-50.4 Gy should be preferred.

This analysis is limited by the retrospective nature of the underlying data and best applies to patients with distal esophageal adenocarcinoma. Efforts were made to minimize the influence of underlying biases and institutional preferences on the results presented, including use of a large, multi-institutional dataset, analysis of CPTTB both as continuous and discretized variable, and incorporation of internal validation and test sets when identifying variables predictive of CPTTB. This potential limitation was also mitigated by the fact that all patients underwent trimodality therapy (including esophagectomy), and there was minimal heterogeneity of radiotherapy dose, fractions of treatment, and the use of concurrent chemotherapy (though the specific chemotherapy agent varied). Further validation of the CPTTB risk score with modern external datasets would also be beneficial. Incorporation of additional cardiac risk factors (e.g. hypertension and hyperlipidemia) could also strengthen our resulting model. Finally, the lack of time-to-event data for post-operative toxicity prevented the use of some statistical approaches.

## Conclusions

CPTTB is an important composite variable and predicts for OS, LOS, and DR60 after trimodality therapy for esophageal cancer. Patients with age ≥ 65 years with post-chemoradiation toxicity are at highest risk for major CPTTB, and strategies to optimize medical management, reduce toxicity from chemoradiation, and improve each patient’s medical status prior to surgery should be strongly considered.

## Data availability statement

The original contributions presented in the study are included in the article/**Supplementary material**. Further inquiries can be directed to the corresponding author.

## Author contributions

All authors listed have made a substantial, direct, and intellectual contribution to the work and approved it for publication.

## References

[1] Eyck BM van Lanschot JJB Hulshof MC van der Wilk BJ Shapiro J van Hagen P. Ten-year outcome of neoadjuvant chemoradiotherapy plus surgery for esophageal cancer: the randomized controlled cross trial. J Clin Oncol (2021) 39:JCO2003614. doi: 10.1200/JCO.20.03614 33891478

[2] Shapiro J Van Lanschot JJB Hulshof MC van Hagen P van Berge Henegouwen MI Wijnhoven BP. Neoadjuvant chemoradiotherapy plus surgery versus surgery alone for oesophageal or junctional cancer (CROSS): long-term results of a randomised controlled trial. Lancet Oncol (2015) 16(9):1090–8. doi: 10.1016/S1470-2045(15)00040-6 26254683

[3] Tepper J Krasna MJ Niedzwiecki D Hollis D Reed CE Goldberg R. Phase III trial of trimodality therapy with cisplatin, fluorouracil, radiotherapy, and surgery compared with surgery alone for esophageal cancer: CALGB 9781. J Clin Oncol (2008) 26(7):1086. doi: 10.1200/JCO.2007.12.9593 18309943 PMC5126644

[4] van Hagen P Hulshof M Van Lanschot J Steyerberg E Henegouwen MVB Wijnhoven B. Preoperative chemoradiotherapy for esophageal or junctional cancer. New Engl J Med (2012) 366(22):2074–84. doi: 10.1056/NEJMoa1112088 22646630

[5] Bhagat R Bronsert MR Juarez-Colunga E Weyant MJ Mitchell JD Glebova NO. Postoperative complications drive unplanned readmissions after esophagectomy for cancer. Ann Thorac Surgery. (2018) 105(5):1476–82. doi: 10.1016/j.athoracsur.2017.12.024 29373825

[6] Chen SY Molena D Stem M Mungo B Lidor AO. Post-discharge complications after esophagectomy account for high readmission rates. World J Gastroenterol (2016) 22(22):5246. doi: 10.3748/wjg.v22.i22.5246 27298567 PMC4893471

[7] Wang Y Yang C-FJ He H Buchan JM Patel DC Liou DZ. Short-term and intermediate-term readmission after esophagectomy. J Thorac Dis (2021) 13(8):4678. doi: 10.21037/jtd-21-637 34527309 PMC8411130

[8] Ma L Li J Shao L Lin D Xiang J. Prolonged postoperative length of stay is associated with poor overall survival after an esophagectomy for esophageal cancer. J Thorac Dis (2015) 7(11):2018. doi: 10.3978/j.issn.2072-1439.2015.11.49 26716041 PMC4669285

[9] Voeten DM van der Werf LR van Sandick JW van Hillegersberg R van Berge Henegouwen MI. Length of hospital stay after uncomplicated esophagectomy. hospital variation shows room for nationwide improvement. Surg Endoscopy (2020) 35:1–14. doi: 10.1007/s00464-020-08103-4 PMC8523439 33104919

[10] Chuong MD Hallemeier CL Jabbour SK Yu J Badiyan S Merrell KW. Improving outcomes for esophageal cancer using proton beam therapy. Int J Radiat Oncol Biol Physics. (2016) 95(1):488–97. doi: 10.1016/j.ijrobp.2015.11.043 PMC10862360 27084662

[11] Garant A Whitaker TJ Spears GM Routman DM Harmsen WS Wilhite TJ. A comparison of patient-reported health-related quality of life during proton versus photon chemoradiation therapy for esophageal cancer. Pract Radiat Oncol (2019) 9(6):410–7. doi: 10.1016/j.prro.2019.07.003 31310815

[12] Lin SH Hobbs BP Verma V Tidwell RS Smith GL Lei X. Randomized phase IIB trial of proton beam therapy versus intensity-modulated radiation therapy for locally advanced esophageal cancer. J Clin Oncol (2020) 38(14):1569–79. doi: 10.1200/JCO.19.02503 PMC7213588 32160096

[13] Garant A Spears G Routman D Whitaker T Liao Z Harmsen W. A multi-institutional analysis of radiation dosimetric predictors of toxicity after trimodality therapy for esophageal cancer. Pract Radiat Oncol (2021) 11:e415–25. doi: 10.1016/j.prro.2021.01.004 33486102

[14] Routman DM Garant A Lester SC Day CN Harmsen WS Sanheuza CT. A comparison of grade 4 lymphopenia with proton versus photon radiation therapy for esophageal cancer. Adv Radiat Oncol (2019) 4(1):63–9. doi: 10.1016/j.adro.2018.09.004 PMC6349594 30706012

[15] Markar S Gronnier C Duhamel A Pasquer A Théreaux J Du Rieu MC. Salvage surgery after chemoradiotherapy in the management of esophageal cancer: Is it a viable therapeutic option? J Clin Oncol (2015) 33(33):3866–73. doi: 10.1200/JCO.2014.59.9092 26195702

[16] Swisher SG Moughan J Komaki RU Ajani JA Wu TT Hofstetter WL. Final results of NRG oncology RTOG 0246: An organ-preserving selective resection strategy in esophageal cancer patients treated with definitive chemoradiation. J Thorac Oncol (2017) 12(2):368–74. doi: 10.1016/j.jtho.2016.10.002 PMC5263046 27729298

[17] Allen S Brown V Prabhu P Scott M Rockall T Preston S. A randomised controlled trial to assess whether prehabilitation improves fitness in patients undergoing neoadjuvant treatment prior to oesophagogastric cancer surgery: Study protocol. BMJ Open (2018) 8(12):e023190. doi: 10.1136/bmjopen-2018-023190 PMC6318540 30580268

[18] Bolger JC Loughney L Tully R Cunningham M Keogh S McCaffrey N. Perioperative prehabilitation and rehabilitation in esophagogastric malignancies: A systematic review. Dis Esophagus (2019) 32(9):doz058. doi: 10.1093/dote/doz058 31206582

[19] Doganay E Moorthy K. Prehabilitation for esophagectomy. J Thorac Disease. (2019) 11(Suppl 5):S632. doi: 10.21037/jtd.2019.02.12 31080639 PMC6503267

[20] Xi M Hallemeier CL Merrell KW Liao Z Murphy MAB Ho L. Recurrence risk stratification after preoperative chemoradiation of esophageal adenocarcinoma. Ann Surgery (2018) 268(2):289–95. doi: 10.1097/SLA.0000000000002352 28628563

[21] Edge SB Byrd DR Carducci MA Compton CC Fritz A Greene F. AJCC cancer staging manual. New York: Springer (2010).

[22] Sudo K Taketa T Correa AM Campagna M-C Wadhwa R Blum MA. Locoregional failure rate after preoperative chemoradiation of esophageal adenocarcinoma and the outcomes of salvage strategies. J Clin Oncol (2013) 31(34):4306. doi: 10.1200/JCO.2013.51.7250 24145339 PMC3837091

[23] Kowalchuk RO Mullikin TC Harmsen WS Rose PS Siontis BL Kim DK. Development and internal validation of a recursive partitioning analysis-based model predictive of pain flare incidence after spine stereotactic body radiation therapy. Pract Radiat Oncol (2022) 12:e269–e277. doi: 10.1016/j.prro.2022.01.011 35151922

[24] Kowalchuk RO Johnson-Tesch BA Marion JT Mullikin T Harmsen W Rose P. Development and assessment of a predictive score for vertebral compression fracture after stereotactic body radiation therapy for spinal metastases. JAMA Oncol (2022) 8:412–9. doi: 10.1001/jamaoncol.2021.7008 PMC8796057 35084429

[25] Kowalchuk RO Shepard MJ Sheehan K Sheehan D Faramand A Niranjan A. Treatment of WHO grade 2 meningiomas with stereotactic radiosurgery: Identification of an optimal group for SRS using RPA. Int J Radiat Oncol Biol Physics. (2021) 110(3):804–14. doi: 10.1016/j.ijrobp.2021.01.048 33548341

[26] Davidson-Pilon C. Lifelines: Survival analysis in python. J Open Source Software (2019) 4(40):1317. doi: 10.21105/joss.01317

[27] Pedregosa F Varoquaux G Gramfort A Michel V Thirion B Grisel O. Scikit-learn: Machine learning in python. J Mach Learn Res (2011) 12:2825–30.

[28] Tosi S. Matplotlib for python developers. Packt Publishing Ltd (2009).

[29] Asaka S Shimakawa T Yamaguchi K Katsube T Usui T Yokomizo H. Postoperative pneumonia after esophagectomy and systemic inflammatory response syndrome. Anticancer Res (2019) 39(2):979–85. doi: 10.21873/anticanres.13202 30711984

[30] Booka E Takeuchi H Nishi T Matsuda S Kaburagi T Fukuda K. The impact of postoperative complications on survivals after esophagectomy for esophageal cancer. Medicine (2015) 94(33). doi: 10.1097/MD.0000000000001369 PMC4616453 26287423

[31] Reichert M Schistek M Uhle F Koch C Bodner J Hecker M. Ivor lewis esophagectomy patients are particularly vulnerable to respiratory impairment-a comparison to major lung resection. Sci Rep (2019) 9(1):1–12. doi: 10.1038/s41598-019-48234-w 31413282 PMC6694108

[32] Pultrum BB Bosch DJ Nijsten MWN Rodgers MGG Groen H Slaets JPJ. Extended esophagectomy in elderly patients with esophageal cancer: Minor effect of age alone in determining the postoperative course and survival. Ann Surg Oncol (2010) 17(6):1572–80. doi: 10.1245/s10434-010-0966-7 PMC2868167 20180031

[33] Ruol A Portale G Zaninotto G Cagol M Cavallin F Castoro C. Results of esophagectomy for esophageal cancer in elderly patients: Age has little influence on outcome and survival. J Thorac Cardiovasc Surgery (2007) 133(5):1186–92. doi: 10.1016/j.jtcvs.2006.12.040 17467427

[34] Ling T Slater J Nookala P Mifflin R Grove R Ly A. Analysis of intensity-modulated radiation therapy (IMRT), proton and 3D conformal radiotherapy (3D-CRT) for reducing perioperative cardiopulmonary complications in esophageal cancer patients. Cancers (2014) 6(4):2356–68. doi: 10.3390/cancers6042356 PMC4276971 25489937

[35] Xu D Li G Li H Jia F. Comparison of IMRT versus 3D-CRT in the treatment of esophagus cancer: A systematic review and meta-analysis. Medicine (2017) 96(31). doi: 10.1097/MD.0000000000007685 PMC5626151 28767597

[36] Zhang A Deek MP Kim S Sayan M Grann A Wagman RT. Vertebral body irradiation during chemoradiation therapy for esophageal cancer contributes to acute bone marrow toxicity. J Gastrointest Oncol (2019) 10(3):513. doi: 10.21037/jgo.2019.01.20 31183202 PMC6534715

[37] Zhang Y Jabbour SK Zhang A Liu B Yue NJ Biswal NC. Proton beam therapy can achieve lower vertebral bone marrow dose than photon beam therapy during chemoradiation therapy of esophageal cancer. Med Dosimetry (2021) 46(3):229–35. doi: 10.1016/j.meddos.2020.12.003 33454170

[38] Minnella EM Awasthi R Loiselle S-E Agnihotram RV Ferri LE Carli F. Effect of exercise and nutrition prehabilitation on functional capacity in esophagogastric cancer surgery: A randomized clinical trial. JAMA Surg (2018) 153(12):1081–9. doi: 10.1001/jamasurg.2018.1645 PMC6583009 30193337

[39] Chan KKW Saluja R Delos Santos K Lien K Shah K Cramarossa G. Neoadjuvant treatments for locally advanced, resectable esophageal cancer: A network meta-analysis. Int J Cancer. (2018) 143(2):430–7. doi: 10.1002/ijc.31312 29441562

[40] Eads JR Weitz M Catalano PJ Gibson MK. A phase II/III study of perioperative nivolumab and ipilimumab in patients (pts) with locoregional esophageal (E) and gastroesophageal junction (GEJ) adenocarcinoma: Results of a safety run-in–a trial of the ECOG-ACRIN cancer research group (EA2174). J Clin Oncol (2021) 39. doi: 10.1200/JCO.2021.39.15_suppl.4064

[41] Li Y Liu H Sun C Yin X Tong J Zhang X. Comparison of clinical efficacy of neoadjuvant chemoradiation therapy between lower and higher radiation doses for carcinoma of the esophagus and gastroesophageal junction: A systematic review. Int J Radiat Oncol Biol Phys (2021) 111(2):405–16. doi: 10.1016/j.ijrobp.2021.04.031 33964352

