# Supplementary material for: Risk stratification of postoperative cardiopulmonary toxicity after trimodality therapy for esophageal cancer
Source: Front Oncol. 2023 Feb 9;13:1081024. doi: 10.3389/fonc.2023.1081024 (PMC9948243; doi:10.3389/fonc.2023.1081024)
Supplement: Supplementary file 1 [file Table_1.docx]

**Supplemental table 1**. Potential predictors of cardiopulmonary total toxicity burden are shown, with statistical significance determined using the Wilcoxon p-value. Variables demonstrating statistical significance are bolded.

| **Variable** | **Wilcoxon p-value** |
| --- | --- |
| Gender | **p=0.013** |
| Age > 65 years | **p<0.001** |
| *Underlying comorbidities*  History of heavy (≥ 6 drinks/day) alcohol use  Coronary artery disease  Diabetes mellitus  COPD | p=0.60  p=0.29  p=0.84  **p=0.023** |
| *Treatment characteristics*  3D radiotherapy  Induction chemotherapy | **p<0.001**  **p=0.028** |
| Clinical T stage ≥ 3 | **p=0.029** |
| *Concurrent chemotherapy*  Cisplatin/5-FU  Carboplatin/paclitaxel  Oxaliplatin/5-FU  5-FU/docetaxel | **p=0.047**  **p<0.001**  p=0.13  **p=0.002** |
| *Post-chemoradiotherapy toxicity*  Median weight loss ≥ 5%  Feeding tube placed  G2+ dysphagia  G2+ esophagitis  G2+ nausea  G2+ fatigue  G2+ anorexia  G2+ hematologic  G3+ hematologic | p=0.95  **p=0.019**  p=0.56  p=0.057  **p<0.001**  p=0.74  p=0.99  **p<0.001**  **p<0.001** |

**Supplemental table 2**. Final feature importance and correlation testing is shown, demonstrating no significant correlation between candidate variables.

| **Variable** | Feature importance | Gender | COPD | Induction chemo | Age | cT stage | Carboplatin/paclitaxel | 5FU/docetaxel | G2+ esophagitis | Feeding tube | G2+ nausea | G3+ heme |
| --- | --- | --- | --- | --- | --- | --- | --- | --- | --- | --- | --- | --- |
| Gender | 0.042 | 1* | 0.002 | 0.054 | -0.007 | -0.038 | -0.069 | -0.0010 | -0.065 | -0.17 | -0.053 | -0.093 |
| COPD | 0.034 | 0.002 | 1* | -0.047 | 0.12 | -0.084 | 0.014 | -0.003 | -0.034 | 0.023 | -0.015 | -0.016 |
| Induction chemo | 0.041 | 0.054 | -0.047 | 1* | -0.036 | 0.088 | -0.21 | -0.071 | -0.12 | 0.017 | -0.072 | -0.15 |
| Age | 0.57 | -0.007 | 0.12 | -0.036 | 1* | -0.016 | 0.083 | -0.073 | 0.014 | 0.036 | 0.076 | 0.053 |
| cT stage | 0.071 | -0.038 | -0.084 | 0.088 | -0.016 | 1* | -0.041 | 0.043 | 0.06 | 0.11 | -0.015 | -0.01 |
| Carboplatin/  paclitaxel | 0.032 | -0.069 | 0.014 | -0.21 | 0.083 | -0.041 | 1* | -0.24 | 0.068 | -0.054 | 0.014 | 0.004 |
| 5FU/docetaxel | 0.029 | -0.010 | -0.003 | -0.071 | -0.073 | 0.043 | -0.24 | 1* | 0.023 | 0.001 | -0.22 | -0.18 |
| G2+ esophagitis | 0.056 | -0.065 | -0.034 | -0.12 | 0.014 | 0.06 | 0.007 | 0.023 | 1* | 0.19 | 0.25 | 0.12 |
| Feeding tube | 0.055 | -0.17 | 0.023 | 0.017 | 0.036 | 0.11 | -0.054 | 0.001 | 0.19 | 1* | 0.16 | 0.047 |
| G2+ nausea | 0.044 | -0.053 | -0.015 | -0.072 | 0.076 | -0.015 | 0.014 | -0.22 | 0.25 | 0.16 | 1* | 0.19 |
| G3+ heme | 0.028 | -0.093 | -0.016 | -0.15 | 0.053 | -0.01 | 0.004 | -0.18 | 0.12 | 0.047 | 0.19 | 1* |

1*: a correlation of 1 is noted for variables compared with the same variable

Abbreviations: COPD = chronic obstructive pulmonary disease; chemo = chemotherapy; cT stage = clinical T stage; 5FU = Fluorouracil; G2+ = at least grade 2; G3+ = at least grade 3

**Supplemental table 3**. The influence of tumor histology, tumor location, radiotherapy dose, and chemotherapy regimen on cardiopulmonary total toxicity burden are shown.

|  | **CPTTB mean (SD)** | **p value** | **No CPTTB** | **Minor CPTTB (10-60)** | **Major CPTTB (≥ 70)** |
| --- | --- | --- | --- | --- | --- |
| **Tumor histology***  Adenocarcinoma (n=530)  SCC (n=40) | 18.2 (34.5)  32.8 (49.7) | 0.040 | 362 (68%)  22 (55%) | 116 (22%)  9 (23%) | 52 (10%)  9 (23%) |
| **Tumor location**  Proximal/middle (n=40)  Distal (n=531) | 33.5 (44.9)  18.1 (34.9) | 0.009 | 20 (50%)  365 (69%) | 10 (25%)  115 (22%) | 10 (25%)  51 (10%) |
| **Radiotherapy dose**  < 50.4 Gy (n=60)  ≥ 50.4 Gy (n=511) | 24.2 (34.3)  18.6 (36.1) | 0.26 | 31 (52%)  354 (69%) | 24 (40%)  101 (20%) | 5 (8%)  56 (11%) |
| **Induction chemotherapy**  Yes (n=139)  No (n=432) | 11.9 (24.9)  21.5 (38.5) | 0.006 | 102 (73%)  283 (66%) | 31 (22%)  94 (22%) | 6 (4%)  55 (13%) |
| **All patients** | 19.2 (35.9) | - | 385 (67%) | 125 (22%) | 61 (11%) |

*One patient did not have tumor histology available for assessment

Abbreviations: CPTTB = cardiopulmonary total toxicity burden; SD = standard deviation; SCC = squamous cell carcinoma

**Supplemental table 4**. Differences of cardiopulmonary total toxicity burden for the difference chemotherapy regimens are tabulated.

| **Chemotherapy** | **Cisplatin/5FU (n=138)** | **Carboplatin/Paclitaxel (n=87)** | **Oxaliplatin/5FU (n=148)** | **Docetaxel/5FU (n=142)** | **DFOX (n=21)** | **Others (n=35)** | **Total (n=571)** | **p value** |
| --- | --- | --- | --- | --- | --- | --- | --- | --- |
| **Mean CPTTB (SD)** | 25.9 (43.0) | 30.2 (41.5) | 14.3 (28.4) | 12.5 (32.1) | 12.4 (25.6) | 16.9 (28.9) | 19.2 (35.9) | <0.001 |

Abbreviations: 5FU = fluorouracil; DFOX = docetaxel, oxaliplatin, and fluorouracil; CPTTB = cardiopulmonary total toxicity burden; SD = standard deviation

**Supplemental table 5**. Adherence to standard heart and lung dose constraints is shown, stratified by treatment modality.

|  | 3D (n=211) | IMRT/Protons (n=360) | Overall (n=571) |
| --- | --- | --- | --- |
| Lung V40 |  |  |  |
| ≤10% | 163 (91.6%) | 290 (94.5%) | 453 (93.4%) |
| >10% | 15 (8.4%) | 17 (5.5%) | 32 (6.6%) |
| Lung V30 |  |  |  |
| ≤15% | 167 (93.8%) | 278 (90.6%) | 445 (91.8%) |
| >15% | 11 (6.2%) | 29 (9.4%) | 40 (8.2%) |
| Lung V20 |  |  |  |
| ≤20% | 152 (85.4%) | 230 (74.9%) | 382 (78.8%) |
| >20% | 26 (14.6%) | 77 (25.1%) | 103 (21.2%) |
| Lung V10 |  |  |  |
| ≤40% | 123 (69.1%) | 268 (87.3%) | 391 (80.6%) |
| >40% | 55 (30.9%) | 39 (12.7%) | 94 (19.4%) |
| Lung V5 |  |  |  |
| ≤50% | 96 (53.9%) | 211 (68.7%) | 307 (63.3%) |
| >50% | 82 (46.1%) | 96 (31.3%) | 178 (36.7%) |
| Lung Mean |  |  |  |
| ≤20 Gy | 178 (100.0%) | 308 (100.0%) | 486 (100.0%) |
| >20 Gy | 0 (0.0%) | 0 (0.0%) | 0 (0.0%) |
| Heart V30 |  |  |  |
| ≤30% | 28 (15.3%) | 210 (68.2%) | 238 (48.5%) |
| >30% | 155 (84.7%) | 98 (31.8%) | 253 (51.5%) |
| Heart Mean |  |  |  |
| ≤30 Gy | 102 (55.7%) | 288 (92.3%) | 390 (78.8%) |
| >30 Gy | 81 (44.3%) | 24 (7.7%) | 105 (21.2%) |

Abbreviations: Vx = volume receiving at least x Gy; 3D = 3D radiotherapy; IMRT = intensity-modulated radiotherapy
